# Incremental Effects of Endocrine and Metabolic Biomarkers and Abdominal Obesity on Cardiovascular Mortality Prediction

**DOI:** 10.1371/journal.pone.0033084

**Published:** 2012-03-16

**Authors:** Harald Jörn Schneider, Henri Wallaschofski, Henry Völzke, Marcello Ricardo Paulista Markus, Marcus Doerr, Stephan B. Felix, Matthias Nauck, Nele Friedrich

**Affiliations:** 1 Institute of Clinical Chemistry and Laboratory Medicine, Ernst Moritz Arndt University Greifswald, Greifswald, Germany; 2 Medizinische Klinik - Innenstadt, Klinikum der Universität, Munich, Germany; 3 Institute for Community Medicine, Ernst Moritz Arndt University Greifswald, Greifswald, Germany; 4 Department of Cardiology, Ernst Moritz Arndt University Greifswald, Greifswald, Germany; Heart Center Munich, Germany

## Abstract

**Background:**

Biomarkers may help clinicians predict cardiovascular risk. We aimed to determine if the addition of endocrine, metabolic, and obesity-associated biomarkers to conventional risk factors improves the prediction of cardiovascular and all-cause mortality.

**Methodology/Principal Findings:**

In a population-based cohort study (the Study of Health in Pomerania) of 3,967 subjects (age 20–80 years) free of cardiovascular disease with a median follow-up of 10.0 years (38,638 person-years), we assessed the predictive value of conventional cardiovascular risk factors and the biomarkers thyrotropin; testosterone (in men only); insulin-like growth factor-1 (IGF-1); hemoglobin A1c (HbA1c); creatinine; high-sensitive C-reactive protein (hsCRP); fibrinogen; urinary albumin-to-creatinine ratio; and waist-to-height ratio (WHtR) on cardiovascular and all-cause death.

During follow-up, we observed 339 all-cause including 103 cardiovascular deaths. In Cox regression models with conventional risk factors, the following biomarkers were retained as significant predictors of cardiovascular death after backward elimination: HbA1c, IGF-1, and hsCRP. IGF-1 and hsCRP were retained as significant predictors of all-cause death.

For cardiovascular death, adding these biomarkers to the conventional risk factors changed the C-statistic from 0.898 to 0.910 (p = 0.02). The net reclassification improvement was 10.6%. For all-cause death, the C-statistic changed from 0.849 to 0.853 (P = 0.09).

**Conclusions/Significance:**

HbA1c, IGF-1, and hsCRP predict cardiovascular death independently of conventional cardiovascular risk factors. These easily assessed endocrine and metabolic biomarkers might improve the ability to predict cardiovascular death.

## Introduction

Scoring systems based on classic risk factors, including sex, age, hypertension, dyslipidemia, and smoking, predict the future risk of cardiovascular events or death [1]–[5]; however, these risk factors explain only part of cardiovascular risk.

Thus, attempts have been made to improve the prediction of cardiovascular risk by adding multiple novel biomarkers to the classic risk factors. These biomarkers have included markers of inflammation, kidney function, cardiac damage, endothelial function, metabolism, and oxidative stress, among others [6]–[11].

However, these novel markers' abilities to improve prediction were mostly disappointing. Many studies failed to detect a clinically relevant improvement in risk prediction [7], [8], [10]. Biomarkers that appeared to improve prediction in some studies [6], [11] did not perform well in other cohorts [7].

Differences in hormone levels are associated with cardiovascular risk. Both elevated and suppressed thyrotropin concentrations have been associated with increased cardiovascular mortality, though not consistently [12]. Low levels of insulin-like growth factor-1 (IGF-1), a metabolic and anabolic hormone as well as a mediator of growth hormone action, have been associated with increased cardiovascular mortality in some studies [13], [14], but not in all [15]. In men, low testosterone levels predict cardiovascular and all-cause mortality [16]–[18]. Hemoglobin A1c (HbA1c) predicted cardiovascular events in several studies, independently of diabetes [19]–[21], although these results were not confirmed by another study [22].

In addition, recent studies suggest that measures of abdominal obesity, such as the waist-to-height ratio (WHtR), are associated with cardiovascular risk, independent of classic cardiovascular risk factors [23], [24]. To our knowledge, no one has studied whether a multimarker approach that includes endocrine and metabolic parameters along with abdominal obesity will improve the predictive value of classic cardiovascular risk factors.

We hypothesized that a comprehensive set of easily assessed biomarkers that reflect different potential pathways of cardiovascular risk, including hormonal imbalance, glucose metabolism, abdominal obesity, inflammation, and kidney damage, adds incrementally to the use of conventional risk factors to predict cardiovascular and all-cause death.

## Materials and Methods

### Subjects

The Study of Health in Pomerania (SHIP) is a longitudinal, representative, population-based cohort study in West Pomerania, a region in northeast Germany. Baseline data was collected from 1997 through 2001. A total of 4,308 subjects (response proportion: 69%) participated [25], [26]. All participants gave written informed consent. The study conformed to the principles of the Declaration of Helsinki, and was approved by the Ethics Committee of the University of Greifswald.

Of the 4,308 participants, 18 pregnant women, 63 with incomplete conventional cardiovascular risk factor information and 260 with history of major cardiovascular events (stroke, myocardial infarction or heart surgery) at baseline were excluded, resulting in a study population of 3,967 subjects. Vital status information was acquired at annual intervals from the time of enrollment through December 2009. Subjects were censored at either death or failure to follow-up. The median duration of follow-up was 10.0 years (25^th^ percentile 9.3; 75^th^ percentile 10.0). Death certificates were coded by a certified nosologist according to the International Classification of Diseases, 10th revision (ICD10). Additionally, two internists (H.W. and M.D.) independently validated the underlying causes of death and performed a joint reading together with a third internist (H.V.) in cases of disagreement. Cardiovascular death included Codes I10 to I79.

### Clinical assessments

Information on age, sex, and medical history was collected with computer-aided personal interviews. Smoking status was assessed by self-report, and subjects were categorized as either current smokers (at least one cigarette per day within the past year) or non-smokers. Anthropometric characteristics were measured according to written, standardized instructions in accordance with World Health Organization standards (WHO 1987). Waist circumference was measured to the nearest 0.1 cm midway between the lower rib margin and the iliac crest in the horizontal plane, using an inelastic tape measure. Blood pressure was measured three times with an appropriate-sized cuff after five minutes of rest in a sitting position, and the mean of the second and third measurement was recorded. The definition of diabetes mellitus was based on self-reported physician's diagnosis or self-reported use of antidiabetic medication in the last seven days. The history of cardiovascular disease (CVD) was based on a self-reported history of myocardial infarction, stroke or cardiac surgery.

### Laboratory measurements

Non-fasting blood samples were drawn from the cubital vein, with the patient in the supine position. A urine sample was collected. The samples were taken between 07:00 a.m. and 04:00 p.m. and analyzed immediately or stored at −80°C until biomarkers could be measured. In addition, internal quality controls were performed at least daily.

Urine samples were stored for a maximum of 2 days until measurement. Serum creatinine levels were determined with the Jaffé method (Hitachi 717; Roche Diagnostics, Germany). The urinary albumin concentration was determined with a Behring Nephelometer (Siemens BN albumin; Siemens Healthcare, Marburg, Germany). Total and high-density lipoprotein (HDL) cholesterol were measured photometrically (Hitachi 704; Roche, Mannheim, Germany).

Serum IGF-1 was determined with automated two-site chemiluminescence immunoassays (Nichols Advantage; Nichols Institute Diagnostica GmbH, Bad Vilbel, Germany). Total testosterone levels were measured with competitive chemiluminescent enzyme immunoassays on an Immulite 2500 analyzer (Siemens Immulite 2500 Total Testosterone, ref. L5KTW, Lot 110; Siemens Healthcare Medical Diagnostics, Bad Nauheim, Germany). Serum thyrotropin levels were measured with immunochemiluminescence (Byk Sangtec Diagnostica GmbH, Frankfurt, Germany). HbA1c levels were determined with high-performance liquid chromatography (Bio-Rad Diamat, Munich, Germany). Plasma fibrinogen concentrations were assayed as described by Clauss (19), using an Electra 1600 analyzer (Instrumentation Laboratory, Barcelona, Spain). HsCRP was determined immunologically on a Behring Nephelometer II with commercially available reagents from Dade Behring (Dade Behring, Eschborn, Germany).

### Statistical analyses

Categorical data were expressed as percentages; continuous data were expressed as medians (25^th^ percentile; 75^th^ percentile). Continuous variables were truncated at the 1^st^ and 99^th^ percentile. Univariate analysis was performed, with χ^2^ testing for categorical variables and Mann–Whitney U-tests for continuous distributions. For regression analyses, skewed variables were log-transformed.

We included the following parameters as conventional risk factors: age (continuous); sex (binary); systolic blood pressure (continuous); antihypertensive medication (binary); HDL cholesterol (continuous); total cholesterol (continuous); diabetes (binary); and current smoking (binary). We assessed the following biomarkers as continuous variables, analyzing the effects of an increase of one standard deviation (SD) from the mean: thyrotropin, IGF-1, testosterone, hsCRP, fibrinogen, HbA1c, creatinine, urinary albumine-to-creatinine ratio (UACR), and WHtR. Additionally, we analyzed cutoffs that were considered clinically useful and were derived from the literature for the following variables: thyrotropin below and above the reference range versus the reference range (0.25–2.12 mIU/l) [27]; IGF-1 below the 10^th^ sex- and age-specific percentile [14]; and testosterone below 10.4 nmol/l [16]. Testosterone was measured in men only, and all analyses that included testosterone were performed in men only.

We proceeded in four steps to assess the prediction of cardiovascular death. First, we performed Cox proportional hazards regression analyses for single biomarkers, unadjusted and adjusted for conventional risk factors, to predict cardiovascular death. Second, we included all biomarkers that were significant after adjustment into a prediction model, using backward elimination with conventional risk factors forced into the model. Third, we used the C-statistic, as described by Pencina et al. [28], to compare the predictive values of conventional risk factors with single, novel risk factors and the models built in the second step [29]. Finally, we classified subjects into groups with low (<2%), moderate (2% to 9%) and high (>9%) risk, based on the recommendations of the European Society of Cardiology [4], and calculated the net reclassification improvement (NRI) for cardiovascular death. The bias-corrected accelerated bootstrap resampling procedure of Efron and Tibshirani was used to obtain 95% confidence intervals for NRIs. Observed risk at 10-years estimated from Kaplan-Meier curve were used to estimate the expected number of subjects who died and who did not die at 10-year follow-up.

In secondary analyses, we repeated the first three steps with all-cause death as outcome. A two-sided p-value of <0.05 was considered statistically significant. Statistical analyses were performed with SAS 9.1 (SAS Institute Inc., Cary, NC, USA).

## Results

### Study sample

Baseline characteristics are shown in table 1. During the 38,638 person-years of follow-up, we observed 339 (8.6%) all-cause deaths (rate per 1,000 person years: 8.8), including 103 (2.6%) deaths due to cardiovascular disease (rate per 1,000 person years: 2.7).

**Table 1:** Baseline characteristics.

Characteristics	N (m/w)	Men	Women
Age, years	1892/2075	50 (36; 63)	48 (35; 61)
Current Smokers, %	1892/2075	38.2	28.6
Diabetes, %	1892/2075	7.7	6.7
Antihypertensive medication, %	1892/2075	26.3	26.7
Systolic blood pressure, mm Hg	1892/2075	141 (129; 153)	126 (114; 142)
Total cholesterol, mg/dl	1892/2075	222 (193; 251)	218 (189; 251)
HDL cholesterol, md/dl	1892/2075	48 (41; 58)	60 (50; 71)
*Biomarkers*			
Waist-to-height ratio	1892/2075	0.54 (0.50; 0.59)	0.50 (0.44; 0.57)
HbA1c, %	1888/2066	5.4 (5.0; 5.8)	5.2 (4.8; 5.6)
IGF-1, ng/ml	1784/1971	133 (104; 170)	135 (102; 174)
Testosterone, nmol/l	1812/0	16.0 (12.7; 20.3)	-
Thyrotropin, mU/l	1872/2046	0.65 (0.43; 0.94)	0.68 (0.44; 1.02)
High-sensitive CRP, mg/l	1745/1917	1.25 (0.65; 2.82)	1.49 (0.68; 3.57)
Fibrinogen, g/l	1887/2067	2.80 (2.43; 3.30)	2.90 (2.54; 3.40)
Serum creatinine, µmol/l	1884/2073	90 (83; 98)	77 (71; 83)
Urinary Albumin-to-Creatinine Ratio, mg/g	1735/1753	7.1 (4.3; 15.0)	9.3 (5.8; 17.3)

N(m/w)  =  number of men and women with data on the respective parameter; HDL  =  high-density lipoprotein; HbA1c  =  hemoglobin A1c; IGF-1  =  Insulin-like growth factor 1; CRP  =  C-reactive protein. Continuous data are expressed as median (25th; 75th percentile); nominal data are given as percentages.

### Prediction of events by single biomarkers

The results of the Cox regression analyses for predicting cardiovascular death with single biomarkers are shown in table 2. WHtR, HBA1c, testosterone below 10.4 nmol/l, hsCRP, and IGF-1 levels below the 10^th^ sex- and age-specific percentile remained significant predictors after adjustment for conventional risk factors.

**Table 2:** Prediction of cardiovascular death by single biomarkers.

	Trans-formation	SD	Total	Events	Unadjusted HR (95%CI)	p	Adjusted HR (95%CI)*	p
WHtR, 1-SD increase	-	0.08	3967	103	2.31 (1.95; 2.75)	<.001	1.41 (1.13; 1.78)	0.003
HbA1c, 1-SD increase	-	0.90%	3954	102	1.64 (1.51; 1.78)	<.001	1.34 (1.13; 1.58)	0.001
IGF-1		57.6 ng/ml	3755	96				
<10th percentile	-				2.32 (1.41; 3.84)	0.001	2.43 (1.46; 4.05)	0.001
1-SD increase	-				0.37 (0.28; 0.50)	<.001	0.76 (0.56; 1.04)	0.09
Testosterone		6.01 nmol/l	1812	58				
<10.4 nmol/l	-				3.47 (1.99; 6.06)	<.001	1.90 (1.05; 3.47)	0.04
1-SD increase	-				0.64 (0.48; 0.86)	0.003	0.89 (0.68; 1.15)	0.37
Thyrotropin		0.77 mU/l	3918	101				
1-SD increase	log				0.81 (0.68; 0.967)	0.02	1.03 (0.86; 1.22)	0.78
<0.25 mU/l (ref.: 0.25–2.12)	-				1.92 (1.11; 3.32)	0.02	1.00 (0.57; 1.75)	1.00
>2.12 mU/l (ref.: 0.25–2.12)	-				no events	-	no events	-
hsCRP, 1-SD increase	log	1.10 mg/l	3662	91	2.07 (1.71; 2.51)	<.001	1.60 (1.29; 1.98)	<.001
Fibrinogen, 1-SD increase	log	0.22 g/l	3954	102	1.82 (1.52; 2.19)	<.001	1.21 (0.98; 1.48)	0.07
Serum creatinine, 1-SD increase	log	0.17 µmol/l	3957	102	1.49 (1.34; 1.66)	<.001	1.11 (0.93; 1.33)	0.27
UACR, 1-SD increase	log	1.06 mg/g	3488	96	1.72 (1.48; 1.99)	<.001	1.09 (0.91; 1.30)	0.36

SD  =  standard deviation; HR  =  hazard ratio; CI  =  confidence interval; WHtR  =  waist-to-height-ratio; HbA1c  =  hemoglobin A1c; IGF-1  =  Insulin-like growth factor 1; hsCRP  =  high-sensitive C-reactive protein; UACR  =  urinary albumin-to-creatinine ratio.

* Models were adjusted for age, sex, systolic blood pressure, high-density lipoprotein cholesterol, total cholesterol, antihypertensive medication, diabetes, and current smoking.

For all-cause death, WHtR, hsCRP, fibrinogen, IGF-1 levels below the 10^th^ percentile, and testosterone below 10.4 nmol/l remained significant predictors after adjustment for conventional risk factors (table 3).

**Table 3:** Prediction of all-cause death by single biomarkers.

	Trans-formation	SD	Total	Events	Unadjusted HR (95%CI)	p	Adjusted HR (95%CI)*	p
WHtR, 1-SD increase	-	0.08	3967	339	1.92 (1.74; 2.12)	<.001	1.19 (1.05; 1.36)	0.008
HbA1c, 1-SD increase	-	0.90%	3954	338	1.44 (1.36; 1.53)	<.001	1.09 (0.98; 1.22)	0.11
IGF-1		57.6 ng/ml	3755	319				
<10th percentile	-				1.47 (1.07; 2.02)	0.02	1.60 (1.16; 2.20)	0.004
1-SD increase	-				0.47 (0.40; 0.55)	<.001	0.87 (0.75; 1.03)	0.10
Testosterone		6.01 nmol/l	1812	204				
<10.4 nmol/l	-				2.24 (1.61; 3.11)	<.001	1.53 (1.08; 2.16)	0.02
1-SD increase	-				0.85 (0.73; 0.98)	0.03	1.01 (0.88; 1.15)	0.94
Thyrotropin		0.77 mU/l	3918	331				
1-SD increase	log				0.85 (0.77; 0.94)	0.002	1.06 (0.96; 1.17)	0.24
<0.25 mU/l (ref.: 0.25–2.12)	-				1.65 (1.20; 2.29)	0.002	0.94 (0.68; 1.31)	0.71
>2.12 mU/l (ref.: 0.25–2.12)	-				1.01 (0.54; 1.90)	0.98	1.06 (0.56; 2.02)	0.85
hsCRP, 1-SD increase	log	1.10 mg/l	3662	315	1.65 (1.49; 1.83)	<.001	1.30 (1.16; 1.46)	<.001
Fibrinogen, 1-SD increase	log	0.22 g/l	3954	337	1.65 (1.49; 1.82)	<.001	1.16 (1.04; 1.30)	0.008
Serum creatinine, 1-SD increase	log	0.17 µmol/l	3957	337	1.41 (1.32; 1.51)	<.001	1.08 (0.97; 1.20)	0.14
UACR, 1-SD increase	log	1.06 mg/g	3488	318	1.51 (1.39; 1.65)	<.001	1.07 (0.97; 1.19)	0.18

SD  =  standard deviation; HR  =  hazard ratio; CI  =  confidence interval; WHtR  =  waist-to-height-ratio; HbA1c  =  hemoglobin A1c; IGF-1  =  Insulin-like growth factor 1; hsCRP  =  high-sensitive C-reactive protein; UACR  =  urinary albumin-to-creatinine ratio.

* Models were adjusted for age, sex, systolic blood pressure, high-density lipoprotein cholesterol, total cholesterol, antihypertensive medication, diabetes, and current smoking.

### Risk prediction with combined biomarkers and risk factors

Table 4 shows the results of backward elimination models that included all significant biomarkers from the previous step with conventional risk factors forced into the model for cardiovascular death. HsCRP, HbA1c, and low IGF-1 remained significant predictors after backward elimination. In men, low testosterone was not retained in the model after backward elimination. Therefore, we did not continue with separate analyses by sex. HsCRP and low IGF-1 remained significant predictors for all-cause death (table 5).

**Table 4:** Prediction of cardiovascular death by backward-elimination.

	HR (95%CI)*	p
*Conventional cardiovascular risk factors*		
age, per year	1.13 (1.10; 1.16)	<.001
Smoker	1.92 (1.13; 3.28)	0.02
Antihypertensive medication	1.15 (0.72; 1.83)	0.56
HDL cholesterol, 1-SD increase	0.77 (0.58; 1.01)	0.06
Female	0.67 (0.42; 1.07)	0.10
Diabetes	1.50 (0.83; 2.71)	0.18
Systolic BP, 1-SD increase	1.18 (0.95; 1.47)	0.13
Total cholesterol, 1-SD increase	0.95 (0.74; 1.20)	0.66
*Biomarkers*		
HbA1c, 1-SD increase	1.28 (1.07; 1.51)	0.005
hsCRP, 1-SD increase	1.50 (1.21; 1.87)	<.001
IGF-1 <10th percentile	2.18 (1.30; 3.67)	0.003

BP  =  blood pressure; SD  =  standard deviation; HR  =  hazard ratio; CI  =  confidence interval; WHtR  =  waist-to-height-ratio; HbA1c  =  hemoglobin A1c; IGF-1  =  Insulin-like growth factor 1; hsCRP  =  high-sensitive C-reactive protein; UACR  =  urinary albumin-to-creatinine ratio; Cox regression analysis with backward elimination with a p-value of 0.05.

* Conventional cardiovascular risk factors were forced into the model.

**Table 5:** Prediction of all-cause death by backward-elimination.

	HR (95%CI)*	p
*Conventional cardiovascular risk factors*		
age, per year	1.10 (1.09; 1.11)	<.001
Smoker	2.17 (1.66; 2.84)	<.001
Antihypertensive medication	1.20 (0.93; 1.54)	0.16
HDL cholesterol, 1-SD increase	0.99 (0.87; 1.12)	0.86
Female	0.51 (0.39; 0.65)	<.001
Diabetes	1.79 (1.34; 2.38)	<.001
Systolic BP, 1-SD increase	1.03 (0.91; 1.16)	0.66
Total cholesterol, 1-SD increase	1.11 (0.98; 1.25)	0.09
*Biomarkers*		
hsCRP, 1-SD increase	1.29 (1.15; 1.45)	<.001
IGF-1 <10th percentile	1.49 (1.08; 2.07)	0.02

BP  =  blood pressure; SD  =  standard deviation; HR  =  hazard ratio; CI  =  confidence interval; WHtR  =  waist-to-height-ratio; HbA1c  =  hemoglobin A1c; IGF-1  =  Insulin-like growth factor 1; hsCRP  =  high-sensitive C-reactive protein; UACR  =  urinary albumin-to-creatinine ratio; Cox regression analysis with backward elimination with a p-value of 0.05.

* Conventional cardiovascular risk factors were forced into the model.

Table 6 shows the C-statistics for cardiovascular death. Adding biomarkers to all conventional risk factors lead to a significant (p  =  0.02) but slight change in the C-statistic for cardiovascular death from 0.898 (95%-CI 0.873–0.923) to 0.910 (95%-CI 0.886–0.934). For all-cause death, the C-statistic changed (p  =  0.09) from 0.849 (95%-CI 0.830–0.868) to 0.853 (95%-CI 0.833–0.872) after biomarkers were included (table 7). The reclassification for cardiovascular death was 10.61% (95%-CI −0.28–24.86) as shown in table 8.

**Table 6:** Improvement of prediction of cardiovascular death by biomarkers.

	C-statistic*of cardiovascular deaths with 95% CI(3602 subjects, 91 events)	Net reclassification improvement with95% CI† (%)
Conventional cardiovascular risk factors only	0.898 (0.873; 0.923)	
*Biomarkers*		
HbA1c, 1-SD increase	0.902 (0.877; 0.927	4.52 (−3.25; 17.39)
hsCRP, 1-SD increase	0.906 (0.881; 0.930)	4.71 (−2.70; 15.92)
IGF-1 <10th percentile	0.902 (0.877; 0.927)	5.80 (−0.66; 21.81)
All biomarkers	0.910 (0.886; 0.934)	10.61 (−0.28; 24.86)

CI  =  confidence interval; SD  =  standard deviation; WHtR  =  waist-to-height-ratio; HbA1c  =  hemoglobin A1c; IGF-1  =  Insulin-like growth factor 1; hsCRP  =  high-sensitive C-reactive protein. Conventional cardiovascular risk factors include age, sex, systolic blood pressure, high-density lipoprotein cholesterol, total cholesterol, antihypertensive medication, diabetes, and current smoking.

* For conventional risk factors only or conventional risk factors + biomarkers.

† The bias-corrected accelerated bootstrap resampling procedure was used to calculate 95% confidence intervals.

**Table 7:** Improvement of prediction of all-cause death by biomarkers.

	C-statistic*of all-causedeath with 95% CI(3602 subjects, 311 events)
Conventional cardiovascular risk factors only	0.849 (0.830; 0.868)
*Biomarkers*	
hsCRP, 1-SD increase	0.852 (0.833; 0.871)
IGF-1 <10th percentile	0.850 (0.830; 0.869)
All biomarkers	0.853 (0.833; 0.872)

CI  =  confidence interval; SD  =  standard deviation; WHtR  =  waist-to-height-ratio; HbA1c  =  hemoglobin A1c; IGF-1  =  Insulin-like growth factor 1; hsCRP  =  high-sensitive C-reactive protein. Conventional risk factors include age, sex, systolic blood pressure, high-density lipoprotein cholesterol, total cholesterol, antihypertensive medication, diabetes, and current smoking.

* For conventional cardiovascular risk factors only or conventional cardiovascular risk factors + biomarkers.

**Table 8:** Reclassification of cardiovascular deaths using the model with both conventional cardiovascular risk factors and biomarkers retained after backward-elimination.

	Model with conventional riskfactors + biomarkers				Reclassified intonew category, %	
Model with conventionalrisk factors only	<2%	2; 9%	>9%	Total, n	Lower	Higher
**<2%**						
Subjects included	2631	58	4	2693	0.0	2.2
Subjects who died of CVD*	25	6	0	31	0.0	19.4
Subjects who did not die of CVD	2606	52	0	2658	0.0	2.0
Observed risk, %†	0.93	10.73	0			
**2%; 9%**						
Subjects included	153	434	58	645	23.7	9.0
Subjects who died of CVD*	4	25	9	38	10.5	23.7
Subjects who did not die of CVD	149	409	49	607	24.5	8.1
Observed risk, %†	2.56	5.80	16.00			
**>9%**						
Subjects included	0	75	189	264	28.4	0.0
Subjects who died of CVD*	0	4	25	29	13.8	0.0
Subjects who did not die of CVD	0	71	164	235	30.2	0.0
Observed risk, %†	0	5.79	13.15			
**Total**						
Subjects included	2784	567	251	3602		
Subjects who died of CVD*	28	36	35	99	**NRI = 10.61%**	
Subjects who did not die of CVD	2756	531	216	3503		
Observed risk, %†	1.02	6.34	13.80			

CVD  =  cardiovascular disease; NRI  =  net reclassification improvement. Conventional cardiovascular risk factors include age, sex, systolic blood pressure, high-density lipoprotein cholesterol, total cholesterol, body-mass-index, antihypertensive medication, lipid-lowering medication, diabetes, and current smoking.

* 10-year Kaplan-Meier estimates were used to estimate the number of subjects who died and who did not die.

† Observed risk at 10-years was estimated from the Kaplan-Meier curve.

## Discussion

In this population-based cohort study, we analyzed whether combining endocrine and metabolic parameters with a marker of abdominal obesity improved the prediction of cardiovascular death with conventional risk factors. We identified a set of additional biomarkers, including low IGF-1, HbA1c, and hsCRP that caused a moderate but significant improvement in the prediction of cardiovascular deaths and correctly reclassified 11% of the population at risk. The predictive value was stronger for cardiovascular than for all-cause death. This result is not surprising because the risk factors selected were aimed at cardiovascular risk and not at other causes of death.

Our results confirm the findings of previous studies showing that hsCRP is a significant predictor of cardiovascular risk in a multimarker approach [7]–[11]. Our results extend the findings of previous reports by assessing the associations of HbA1c, and low IGF-1 in this context.

HbA1c was included in one multimarker study, but only in subjects with diabetes [9]. Several studies focusing on HbA1c found a predictive role independent of diabetes [19–219]. We confirmed this role in combination with other biomarkers.

Testosterone in men and thyrotropin did not improve prediction. Although low levels of testosterone have been shown to predict mortality even after adjustment for cardiovascular risk factors [18], the fact that the associations became weaker after adjustment in our study suggests that the effects of testosterone are mediated by cardiovascular risk factors. This result is consistent with previous studies showing that testosterone is associated with cardiovascular risk factors [30], [31]. Similar associations may play a role in the results found for thyrotropin [32].

Low IGF-1 was a significant predictor. Experimental data show IGF-1's protective effects against systemic inflammation, insulin resistance, and free fatty acid production, all of which are consequences of obesity [33]. IGF-1 remained a significant predictor among parameters that reflect these different pathways. This finding suggests that additional parameters are involved in the association between IGF-1 and cardiovascular death. Possibly, the effects of IGF-1 on endothelial function and cardiac growth and function [34], [35] or other, unknown factors play a part.

Previous studies have shown that WHtR is a strong and linear predictor of cardiovascular risk, compared with other measures of abdominal and overall obesity [23], [24], [36] whereas BMI shows a U-shaped association with cardiovascular risk and mortality [37]–[39]. WHtR was not significant in this multimarker approach. Most likely, this is due to the fact that abdominal obesity, as measured by the WHtR, increases risk by promoting conventional cardiovascular risk factors.

The identification of biomarkers to improve the ability to predict cardiovascular risk has been a focus of interest in recent years. Our results show a modest improvement in prediction compared with other studies [7]–[10]. The inclusion of IGF-1 and HbA1c may have contributed to this effect. Other studies found higher incremental effects of biomarkers than our study [6], [11]. Selection of high-risk patients or different outcomes might play a part in these findings.

It has been shown that biomarkers can result in relevant reclassification, even in the absence of significant increases in C-statistics [9]. In our study, 11% of subjects were correctly net reclassified by the new biomarkers. Generally, one can expect that the lower the costs for a biomarker are the greater is the willingness to accept small increments in discrimination.

We selected biomarkers that can be easily obtained in every-day clinical practice. Therefore, the increment in our study, though only moderate, might be relevant if validated, given the low cost and effort associated with biomarker assessment – HbA1c is a routine laboratory parameter and hsCRP is increasingly used in risk assessment. IGF-1 is mainly used as marker of pathological changes in growth hormone secretion in an endocrine setting. All of the biomarkers we measured can be assessed clinically or in blood and do not require fasting.

Several limitations need to be addressed. First, because we aimed to explore new biomarkers, our findings need validation in additional cohorts. We did not test other potentially relevant markers, such as troponin or B-type natriuretic peptide. We expect that these factors are more relevant in populations with higher baseline risks of cardiac damage than our sample had. As in any study of risk prediction, multiple testing is an issue, although we tried to minimize the number of tests we administered. In addition, we only assessed cardiovascular and all-cause mortality. We do not know if our findings are generalizable to other cardiovascular events.

In summary, we found a set of biomarkers that improve the prediction of cardiovascular death. The effort required to assess these parameters is low. Thus, the increase in the C-statistic, though only moderate, and the net reclassification of 11% could have a clinical benefit, once validated.
